# Enhanced Therapeutic Treatment of Colorectal Cancer Using Surface-Modified Nanoporous Acupuncture Needles

**DOI:** 10.1038/s41598-017-11213-0

**Published:** 2017-10-10

**Authors:** Bo Ram Lee, Hye-Rim Kim, Eun-Sook Choi, Jung-Hoon Cho, Nam-Jun Kim, Jung-Hee Kim, Kyeong-Min Lee, Abdul Razzaq, Hansaem Choi, Yunju Hwang, Craig A. Grimes, Bong-Hyo Lee, Eunjoo Kim, Su-Il In

**Affiliations:** 1 Division of Nano & Energy Convergence Research, DGIST (Daegu Gyeongbuk Institute of Science and Technology), 333 Techno Jungang-daero, Hyeonpung-myeon, Dalseong-gun, Daegu, 42988 Republic of Korea; 2 Energy Science & Engineering DGIST, 333 Techno Jungang-daero, Hyeonpung-myeon, Dalseong-gun, Daegu, 42988 Republic of Korea; 3 College of Korean Medicine, Daegu Haany University, 136 Shincheondong-ro, Suseong-Gu, Daegu, 42158 Republic of Korea; 4 Flux Photon Corporation, 116 Donmoor Court, Garner, NC 27529 United States

## Abstract

Acupuncture originated within the auspices of Oriental medicine, and today is used as an alternative method for treating various diseases and symptoms. The physiological mechanisms of acupuncture appear to involve the release of endogenous opiates and neurotransmitters, with the signals mediating through electrical stimulation of the central nervous system (CNS). Earlier we reported a nanoporous stainless steel acupuncture needle with enhanced therapeutic properties, evaluated by electrophysiological and behavioral responses in Sprague-Dawley (SD) rats. Herein, we investigate molecular changes in colorectal cancer (CRC) rats by acupuncture treatment using the nanoporous needles. Treatment at acupoint HT7 is found most effective at reducing average tumor size, β-catenin expression levels, and the number of aberrant crypt foci in the colon endothelium. Surface modification of acupuncture needles further enhances the therapeutic effects of acupuncture treatment in CRC rats.

## Introduction

Worldwide, cancer is now considered the second leading cause of death^1–4^. Consequently, the development of an effective and comfortable medical procedure for cancer treatment is of global interest. During last few decades, acupuncture treatment has been proven as a promising treatment for alleviating chronic health problems such as ischaemia and toxic protein aggregation in the brain^5,6^, gastrointestinal^7^, treatment of inflammatory bowel disease^8^, reducing pain, fatigue, anxiety, nausea and vomiting, urinary bladder cancer treatment^9^ and hot flashes that are commonly associated with cancer treatments^9–12^. All these results provide molecular level-evidence of the therapeutic effects induced by acupuncture. While the physiological mechanisms behind the analgesic effects of acupuncture are unclear, it is believed that it proceeds by release of endogenous opiates and neurotransmitters at the systemic and local tissue levels^12^. It has been suggested that the effect of acupuncture are mediated through electrical stimulation of the central nervous system (CNS)^13^, leading to reduced tissue inflammation and fibrosis via increased influx of anti-inflammatory cytokines and efflux of toxins, cellular debris, and proteins^14,15^.

Acupuncture therapies/treatment are commonly practiced by manual manipulation of acupuncture needles at certain points (acupoints) as dependent upon the nature of the disease. Moreover, a few studies have reported enhanced acupuncture efficacy via modification of diverse parameters such as acupuncture needle diameter^16^, depth of insertion^17^, and needle surface^18^. Inspired by such intriguing results, recently we reported fabrication of nanoporous acupuncture needle (PN) with hierarchical micro/nano-scale pores upon their surface that, in turn, provide higher surface areas than conventional acupuncture needle (CN)^19^. The PN was tested *in vivo* for electrophysiological and behavioral responses of Wistar rats^19^, resulting in improved therapeutic effects without loss of comfort.

Building upon our earlier results^19^, herein we investigate the effects of acupuncture treatment during both the initiation and maturation stages of colorectal cancer (CRC) in rats. The number of aberrant crypt foci (ACF) at the initiation stage and progression of adenocarcinoma and tumor growth at maturation stages was tracked, while differentially expressed genes (DEGs) were used to investigate the effects at the initiation stage. The PN is found more effective in reversing changes in gene expression and tumor growth than CN, suggesting the ability to augment therapeutic effects in cancer treatment.

## Results

### Nanoporous acupuncture needle (PN)

**Figure 1:**
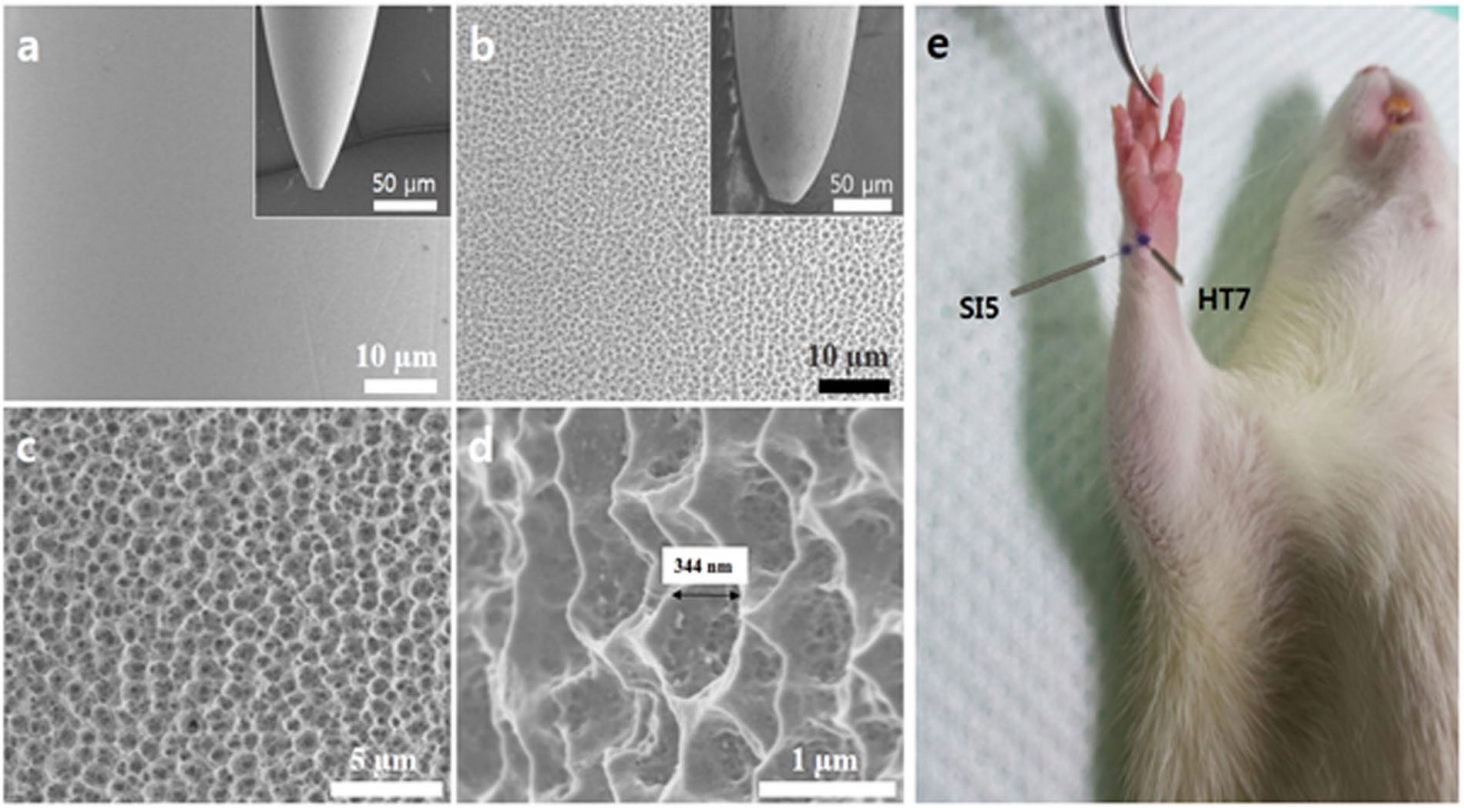
Surface images of (**a**) Conventional acupuncture needle (CN) and, (**b**) The nanoporous acupuncture needle (PN) with its (**c** and **d**) high resolution images. (**e**) Acupoints of rats used in this study.

A stainless steel nanoporous acupuncture needle (PN), prepared according to our recent study^19^, is used and the effects compared to those of a conventional stainless steel acupuncture needle (CN). Surface images of both a CN and PN, obtained using field emission scanning electron microscopy (FE-SEM), are shown in Fig. 1. The CN, initially having a smooth surface, Fig. 1a, after anodization obtains a micro-nanoscale porous surface topology of high surface area, see Fig. 1b–d. Elemental analysis of the needles is tabulated in Supplementary Table S1, which clearly indicates that no impurities are incorporated within the needle during the anodization. PN surface areas, fabricated at various anodization voltages, determined using dye desorption measurements and Beer-Lambert’s Law, are shown in Supplementary Figure S1. The PN fabricated using an anodization voltage of 20 V shows an estimated surface area of 1.03 m^2^∙g^−1^, a maximum value approximately twenty-five times higher than CN (0.04 m^2^∙g^−1^). Due to its optimal surface area 20 V PN is employed for the majority of investigations within the present work.

### Colorectal cancer (CRC) rats model for acupuncture treatment

To induce CRC in rats, azoxymethane (AOM)^20,21^ is administered according to the experimental scheme provided in Table 1. The progress of CRC by AOM to initiation (I) and maturation (M) stages are presented in Supplementary Figure S2. Cancerous polyps are observed at the initiation stage, while severe adenocarcinomas appeared at the maturation stage. From one week following the final AOM injection, acupuncture was performed every day for 4 weeks; Fig. 1e shows the acupoints used in the study. For the initiation stage groups I1–I6 acupoints HT7^22^ and SI5^23^ were used, while HT7 was used for the maturation stage groups M7–M10; rats that did not receive acupuncture treatment were handled for the same amount of time as those receiving treatment.

**Table 1:** Animals and acupuncture treatments.

Stage	Group	Total No. of animals	Age at AOM treatment (week)	Age at acupuncture treatment (week)	Needle^a)^	Acupoint	Age at sacrifice (week)
Initiation	I1	3	N.T.^b)^	N.T.	—	—	10
	I2	3	4–5	N.T.	—	—	
	I3	4	4–5	6–9	CN	HT7	
	I4	4	4–5	6–9	CN	SI5	
	I5	4	4–5	6–9	PN	HT7	
	I6	4	4–5	6–9	PN	SI5	
Maturation	M7	6	N.T.	N.T.	—	—	49
	M8	6	4–5	N.T.	—	—	
	M9	6	4–5	45–48	CN	HT7	
	M10	6	4–5	45–48	PN	HT7	

^a)^CN, conventional acupuncture needle; PN, nanoporous acupuncture needle.

^b)^N.T., no treatment.

### Early stage CRC gene expression analysis

For the initiation stage groups I1–I6, gene expression in the distal colon was analyzed using next generation sequencing (NGS)^24^. Supplementary Table S2 shows, compared to I1, the differentially expressed genes (DEGs) in I2–I6. Pathways that were significantly changed (*p* < 0.05) in each group compared to I1, determined using the iPathwayGuide program^25^, are listed in Supplementary Table S3. Based on statistically significant fold changes, the number of DEGs selected in I2 was 46. In I3 and I4, 21 and 69 genes were, respectively, deregulated, and in I5 and I6, 11 and 157 genes were respectively selected as DEGs. In I5 no pathways were determined to be significantly altered by AOM exposure; in I4 and I6, only adipocytokine-related pathways changed significantly, while in I3 three pathways showed significant changes. Although more DEGs were selected in I4 and I6 than in I2, key molecules involved in altered pathways of I4 and I6 could be recovered by CN or PN treatment at acupoint SI5, as confirmed by pathway analysis. Taken as a whole, the results suggest that CN or PN acupuncture treatment can be used to recover pathway alterations due to AOM exposure, and PN treatment at HT7 is the most effective for DEG reverse-expression.

**Figure 2:**
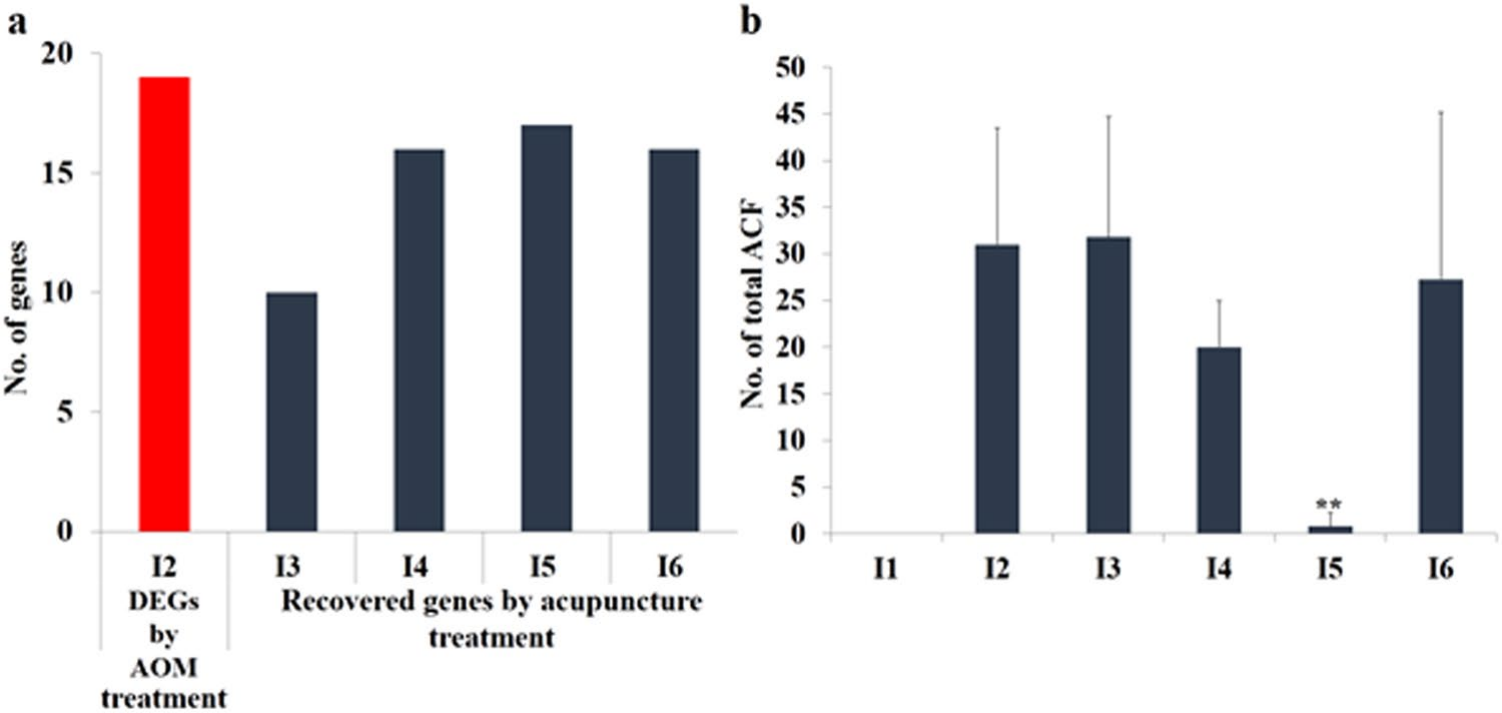
(**a**) The number of genes recovered by acupuncture treatment in each initiation group (I3–I6) for the selected DEGs after AOM exposure in I2. The total number of DEGs in I2, relative to I1, was 19. (**b**) Average number of ACF found in each group. The experimental conditions of initiation groups I2–I6, all at early stage CRC, are as follow: I2 = Positive control (AOM Injection only); I3 = CN treatment at HT7 point; I4 = CN treatment at SI5 point; I5 = PN treatment at HT7 point; and I6 = PN treatment at SI5 point.

To identify the molecules that were recovered by acupuncture treatment, the 46 DEGs in I2 were analyzed using qPCR. Among these, the results for 19 genes were matched to those of the NGS result (*p* < 0.05). Supplementary Table S4 shows the expression level of 19 DEGs relative to I1 or I2, expressed as -ΔΔC_T_^26^. If the effect was reversed by acupuncture treatment, -ΔΔC_T_,_G1_ and -ΔΔC_T_,_G2_ would have opposite signs. In Fig. 2a, the total number of molecules whose expression was reversed was 10, 16, 17, and 16 for I3, I4, I5, and I6, respectively, indicating that at the molecular level CN and PN reversed the adverse effects of AOM exposure. I5 showed the most prominent response to acupuncture treatment, while the least effect was observed in I3, a result consistent with the pathway analysis shown in Supplementary Table S4.

### Nanoporous acupuncture needle (PN) treatment effect on Aberrant crypt foci (ACF) formation

Aberrant crypt foci (ACF) are microscopic lesions that are known to precede the development of dysplasia or adenoma in the colon and are considered to be the earliest preneoplastic lesions in colon carcinogenesis^27^. ACF increase in crypt multiplicity with time, and thus are accepted as a predictor of tumor progression^28^. Supplementary Figure S3 shows representative images of aberrant crypts (ACs) with 1, 2, 3, and ≥ 4 multiplicities. The number of ACF found in the distal colon was categorized according to AC multiplicity and the total number of ACF in each rat colon was determined (Fig. 2b and Supplementary Table S5). The mean number of total ACF in I2 was 31.0 ± 12.5. The ACF count dramatically decreased to 0.8 ± 1.5 ACF/colon in I5, demonstrating that PN at HT7 acupuncture significantly inhibits AOM-induced colonic ACF formation in rats. The reduced ACF number is, presumably, closely related to the recovered expression of genes altered by AOM exposure.

### Long-term acupuncture treatment effects on tumor mass, number and size

To analyze the long-term effects of acupuncture on AOM-induced CRC, maturation groups M7 to M10 were prepared and acupuncture was performed at the HT7 acupoint beginning 40 weeks after the last AOM injection. As shown in Supplementary Tables 6 to 9, the number of discovered polyps was 12, 25, 22, and 17 in M7, M8, M9, and M10, respectively. We sampled all of the polyps from M7 to M10, staining the tissue section with hematoxylin-eosin. In M7, all polyps were normal without cancerous cells, but in M8, M9, and M10, respectively, 6, 4, and 4 rats were harboring at least one tumor. The number of polyps diagnosed as tumors was 10, 8, and 6 from M8, M9, and M10 respectively.

**Figure 3:**
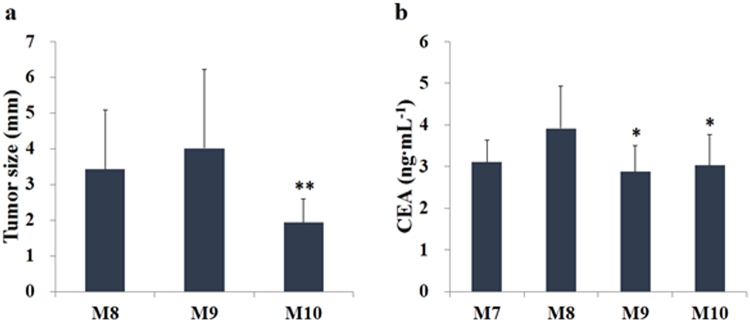
(**a**) Average size of tumors identified in the colon of animals from each experimental group. (**b**) Expression of the circulating colorectal cancer biomarker, CEA. All animals were treated by acupuncture at acupoint HT7 every day for 40 weeks after the last azoxymethane (AOM) injection. M8, positive control; M9, treated with the conventional acupuncture needle (CN); M10, treated with the nanoporous acupuncture needle (PN). Maturation groups M7–M10, all at late stage CRC, have experimental conditions: M7 = Negative control (no treatment); M8 = Positive control (AOM injection only); M9 = CN treatment at HT7 point; and M10 = PN treatment at HT7 point.

Figure 3a shows the average tumor size in each group. Interestingly, the average tumor size observed in M10 was significantly decreased from that of M8. We hypothesize that the acupuncture treatment is connected to the reduction seen in final tumor number and size. To help elucidate cause and effect we analyzed the serum level of carcinoembryo antigen (CEA), a biomarker for CRC^29^, in M7–M10. Figure 3b shows a significant decrease in the average CEA concentration in M9 and M10 compared to M8, providing further evidence as to the efficacy of acupuncture treatment.

### Acupuncture treatment effects on β-catenin expression

In colon cancer, β-catenin expression has been reported to be an important indicator of the cancerous state of the colon endothelium^30,31^. Conducting immunohistochemistry (IHC) for β-catenin in the distal colon of I2, we observed weak expression of β-catenin in the mucosal and submucosal layers of the crypts (Supplementary Figure S4). Animals from I3 and I4 express minimal levels of β-catenin, however in animal groups I5 and I6 the expression levels appear similar to those from I1.

**Figure 4:**
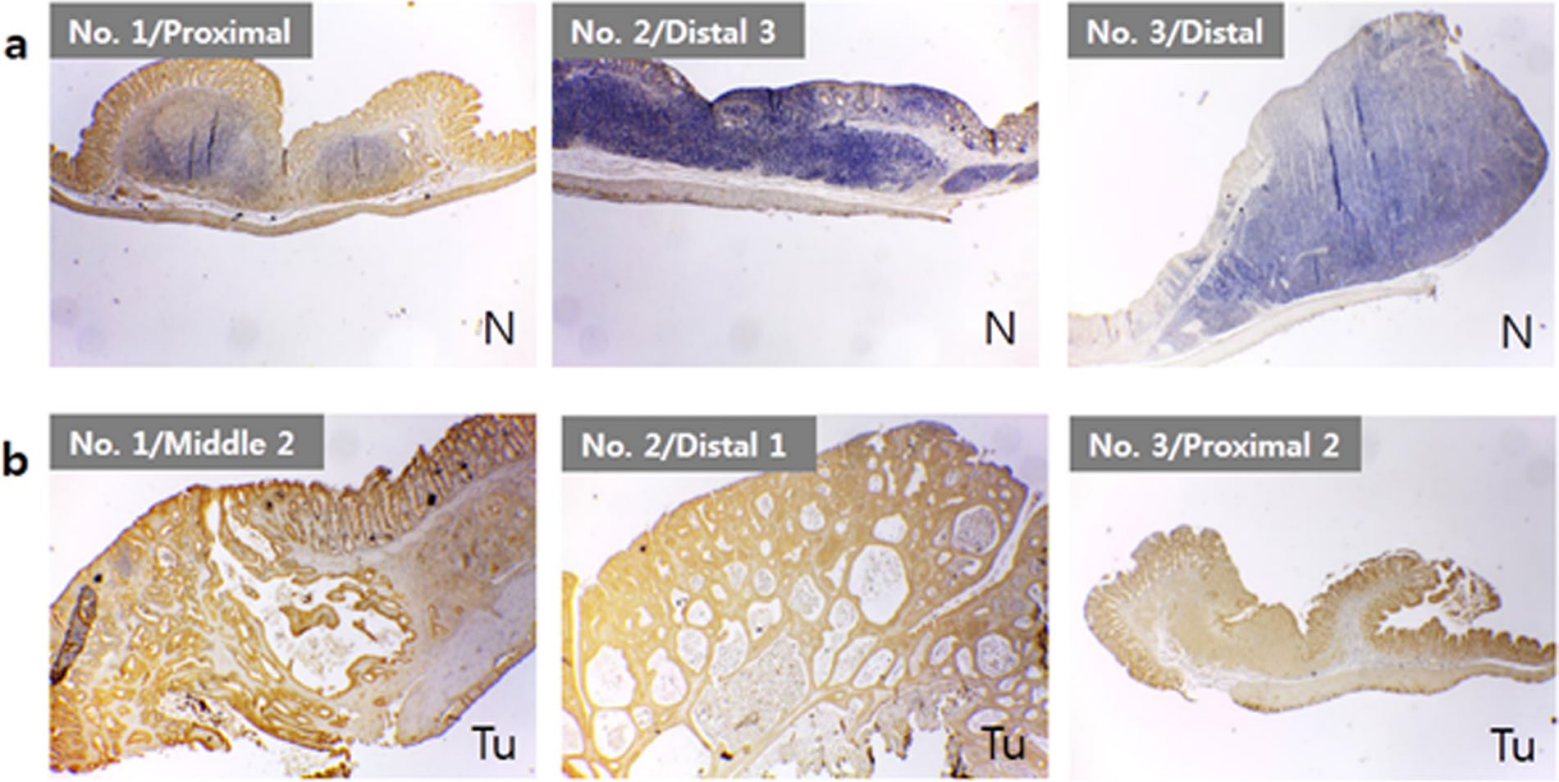
β-Catenin expression in polyps found in colons of maturation stage of CRC (positive control group, M8). (**a**) Polyps categorized as normal tissues, and (**b**) Cancerous tumors. Images magnified at ×40. “No. 1” represents the respective region of animal #1, similarly 2 and 3 stands for animal #2 and animal #3, respectively. “N” and “Tu” stands for mean normal tissue and tumor diagnosed tissue, respectively. See Supplementary Table S7 for sample numbering and diagnostic information.

**Figure 5:**
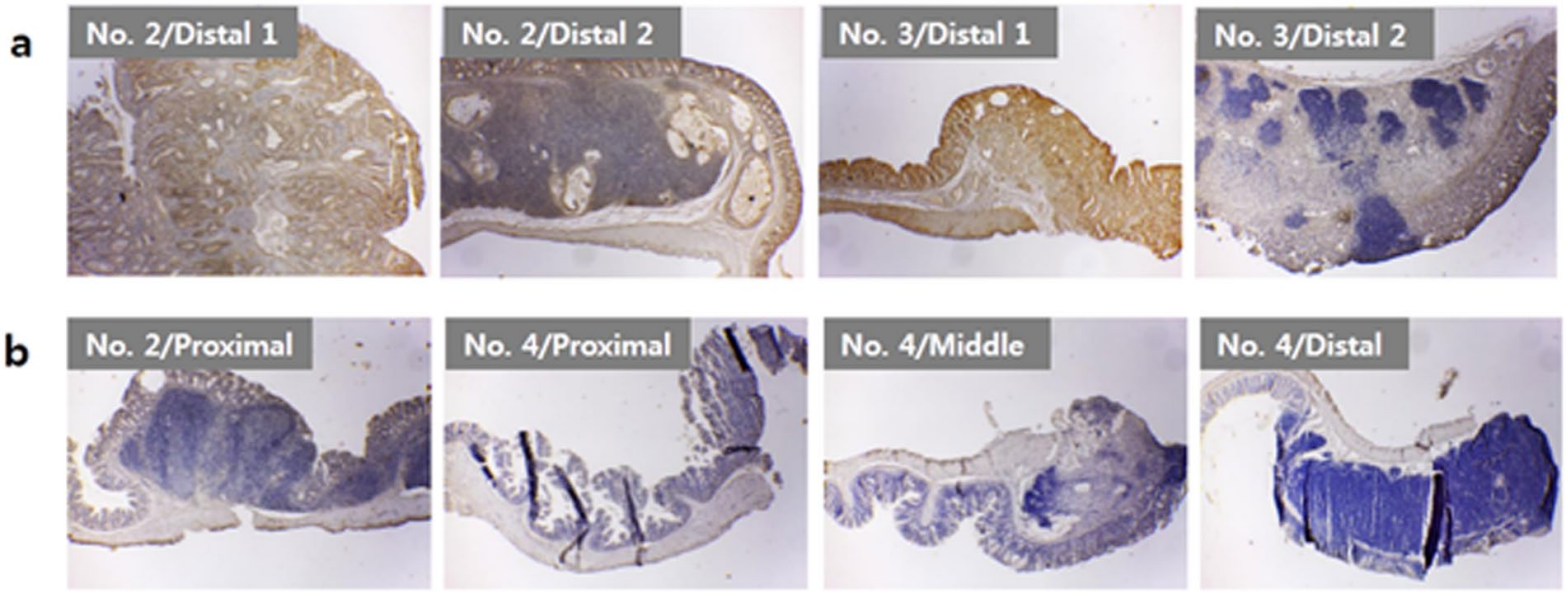
β-Catenin expression in cancerous polyps found in colons following acupuncture treatment. (**a**) M9 (acupuncture treatment using CN) and (**b**) M10 (acupuncture treatment using PN). All Images magnified at ×40. Sample information, taken from various animals within group M9 and M10, are tabulated in Supplementary Tables S8 and S9, respectively. “No. 2” represents the respective colon region of animal #2, similarly 3 and 4 stands for animal #3 and animal #4, respectively.

Figure 4 shows β-catenin distribution in polyps from group M8 animals where, from the same colon, a higher level of β-catenin was expressed by the tumors (Fig. 4b) than the normal polyps (Fig. 4a). In Fig. 5, β-catenin levels in tumors from M9 and M10 group animals are compared. The expression in M9 varied from sample to sample, even though all samples, Fig. 5a, were diagnosed as cancerous tissues. The expression of β-catenin was dramatically reduced in cancerous polyps from M10 animals, Fig. 5b.

To confirm the IHC results, we prepared protein extracts from non-polyp-background tissues taken from the distal tubules of each animal, analyzing β-catenin levels using enzyme-linked immunological assay (ELISA). Supplementary Table S10 shows the β-catenin concentration normalized to the amount of total protein (pg β-catenin/mg protein) in the colon extract. The average concentration of β-catenin was highest in M8 animals, where the concentration was almost ten to hundred times higher than M7 animals. For M9, they varied by almost 2-orders between animals within the group. However, the levels in M10 animals were reduced to almost the same level as M7, and were significantly different from M8 (*p* = 0.0048). The noticeably lower level in M10 was consistent with the IHC data. The results suggest PN treatment promotes a regression of average tumor mass in the maturation stage of CRC.

## Discussion

While cancer treatment using conventional acupuncture needle (CN) exhibited reverse expressions for the molecular deregulation in CRC rats, treatment using nanoporous acupuncture needle (PN) showed significantly enhanced performance as confirmed from DEGs like ATF3, COL5A3, and SLC1A3 (Supplementary Table S4), previously reported as cancer-related biomarkers^27,32,33^. The ACF numbers, commonly accepted as a predictor of tumor progression^28,34^, for the rats in groups I1 to I4 (see Supplementary Table S5) are abated when CN treatment is employed 2 weeks after the first AOM administration, indicating impediment of colonic preneoplastic lesions. PN treatment manifest a significant decrease of ACF numbers, as indicated in groups I5 and I6.

At the maturation stage of CRC, CEA levels are observed to decrease in more or less similar pattern for both acupuncture treatments i.e. using CN (Fig. 3b for group M9) and PN (Fig. 3b for group M10). However, the average tumor mass in the distal tubule is reduced significantly for PN acupuncture treated rats (Fig. 3a group M10). The reduced tumor size may be due to effects other than the acupuncture treatment, such as difference in tumor mass at the start of the treatment by long-term exposure of AOM. However, the decreased levels of distal tubule β-catenin expression at the maturation stage strongly supports the enhanced therapeutic effect of PN over CN (Fig. 5b for group M10). It is well documented, in colon cancer, β-catenin expression is an important indicator of the cancerous state of the colon endothelium^30,31,35^. Hence, the dramatic decrease of β-catenin expression levels for group M10 is indicative of the PN effect. Further, the quantitative determination of β-catenin expression in non-polyp tissues by ELISA indicates the selective decrease of β-catenin in PN-treated group M10 (Supplementary Table S10).

The commonly accepted operational mechanism of acupuncture is that an electrical signal is transferred from the acupoint of the body, such as the hand, foot, or skin, to the disease origin. We previously reported that this electrical signal is enhanced when acupuncture is performed using a PN^19^. Supplementary Figure S5 shows the fitted Nyquist plots for both CN and PN. The similar shapes indicate no surface effects due to the electrolyte, while the substantial decrease of the semicircle diameter for PN indicates decreased charge transfer resistance due to increased PN surface area. While the exact mechanism between electric signal and alteration of molecular expression has not been clearly identified, perhaps the enhanced effect of PN is due the elevated strength of electric signals from PN.

In summary, we show that acupuncture performed using a PN improves therapeutic effects of CRC treatment in rats. The results provide exciting evidence indicating the utility of acupuncture for disease treatment.

## Methods

### Preparation of nanoporous acupuncture needle (PN)

The conventional stainless steel acupuncture needle (CN) used in this investigation were obtained from Dongbang Acupuncture Inc., Republic of Korea, with the dimensions of 8 mm length and 0.18 mm diameter. The PN was prepared by electrochemical anodization as reported previously^19^. Briefly, the electrochemical anodization of sequentially washed CN in acetone, ethanol and deionized (DI) water was carried out in a two-electrode cell, with carbon paper (Carbon and Fuel Cell, CNL, Republic of Korea) as the counter electrode and CN as a working electrode. The electrolyte consisted of 0.2 wt. % (weight percent) NH_4_F (98.0%, American Chemical Society (ACS) reagent, Alfa Aesar, United States) and 2.0 vol. % (volume percent) DI water in ethylene glycol. The anodization was performed at 20 V for 30 min. The anodized needle was rinsed with acetone, ethanol and DI water and then dried in a nitrogen gas stream.

### Characterization of acupuncture needles

Surface images of both CN and PN were obtained using a Field Emission Scanning Electron Microscope (FE-SEM, Hitachi S-4800) operating at 3 kV. Elemental analysis of the CN and PN were done using Energy Dispersive Spectroscopy (EDS, Bruker Co.) built in FE-SEM machine. The absorption spectra for the dye solutions desorbed from CN and PN samples were obtained using a Cary series UV-visible near IR spectrophotometer (Agilent Technologies) in the range of 550–750 nm wavelength. Electrochemical impedance spectroscopy (EIS) spectra were obtained using a Bio Logics SAS (Model VSP-1158) three-electrode workstation. The three electrode cell was comprised of a platinum (Pt) wire counter electrode, acupuncture needle (CN or PN) working electrode, and Ag/AgCl reference electrode, all immersed in saline solution (0.9 g NaCl in 100 ml DI water) purchased from JW-Pharma, Republic of Korea and used as obtained. EC Lab software was used to operate the system over a frequency range of 100 kHz to 1 MHz.

### Development of the rat colorectal cancer (CRC) model

Three-week old male SD rats were obtained from Central Laboratories Animal Inc. (Seoul, Republic of Korea). Animals were maintained in the DGIST Animal Laboratory (Daegu, Republic of Korea) in accordance with the Institutional Animal Care Guidelines. The Animal Care and Use Committee of DGIST approved all animal protocols. After one week of acclimatization, rats were separated into ten groups as shown in Table 1. With the exception of the negative control groups I1 and M7, all rats received 15 mg∙kg^−1^ AOM (Sigma-Aldrich, St. Louis, MO, USA) via intraperitoneal injection once a week for two weeks. The animal experiments were performed in accordance with guideline regulation of the standards of Use Committee and National Institutes of Health (NIH) guidelines and the Institutional Animal Care and Use Committee (IACUC) at the Daegu Haany University. All experimental protocols were approved by the Daegu Haany University IACUC for this specific study (animal use protocol number DHU2014-051) including animal care, housing, auto-controlled housing facility (temperature and humidity) and sanitization.

### Acupuncture treatment

With the exception of the positive control groups (I2 and M8), all rats that received AOM were treated with either CN or PN acupuncture. Acupuncture treatment was carried out bilaterally for 1 min at each acupoint. Negative control groups (I1 and M7) received the same treatment as the acupuncture rats but without needle stimulation. The HT7 acupoints are located on the transverse crease of the wrist of the forepaw, radial to the tendon of the muscle flexor carpi ulnaris; SI5 acupoints are located on the posteromedial aspect of the wrist, in the depression between the triquetrum bone and the ulnar styloid process^36^. Needle was vertically inserted to a depth of 2–3 mm. While the needle was inserted and withdrawn stimulation was produced by bidirectional twisting of the needle at a frequency of twice per second for a total of 2 s. Acupuncture treatment was performed with the animals awake, without anesthetization, under a slight movement restriction. Animals received daily handling for 2–3 min to minimize the stress from the movement restriction. Rats received acupuncture treatments once a day for 4 weeks, starting one week after the last AOM injection for the treatment at initiation stage, and starting forty weeks after the last AOM injection for the treatment at maturation stages of cancer.

### RNA isolation and NGS sequencing

Rats from the I1 to I6 groups were sacrificed for colon resection by CO_2_ inhalation one week after their last acupuncture treatment. Colons were opened longitudinally and rinsed with phosphate buffered saline (PBS). An approximately 1 cm wide piece of tissue was removed from the internal end of the distal tubule. Half of each piece of tissue sample was immersed in TRIzol® reagent (Thermo Fisher Scientific, Waltham, MA, United States) for total RNA isolation, and another piece was imbedded in paraffin block for histological and IHC analysis. Total RNA was isolated from the tissue using a Hybrid-R™ RNA Kit (GeneAll®™, Republic of Korea), according to manufacturer’s instructions. RNA purity was determined by assaying 1 µL of total RNA extract on a NanoDrop 8000 spectrophotometer. Total RNA integrity was checked using an Agilent Technologies 2100 Bioanalyzer that generated an RNA Integrity Number (RIN) value, and RNA with RIN above 8 was used for sequencing analysis. mRNA sequencing libraries were prepared according to manufacturer’s instructions (Illumina TruSeq Stranded mRNA Library Prep kit, San Diego, CA, United States). mRNA was purified and fragmented from total RNA (1 μg) using poly-T oligo-attached magnetic beads using two rounds of purification. Cleaved RNA fragments primed with random hexamers were reverse transcribed into first strand cDNA using reverse transcriptase, random primers, dUTP in place of dTTP (incorporation of dUTP quenches the second strand during amplification because the polymerase does not incorporate past this nucleotide). cDNA fragments then had the addition of a single ‘A’ base and subsequent ligation of the adapter. Products were purified and enriched by PCR to create the final strand specific cDNA library. The quality of the amplified libraries was verified by capillary electrophoresis (Bioanalyzer, Agilent). After qPCR using SYBR Green PCR Master Mix (Applied Biosystems), we combined libraries that index tagged in equimolar amounts in the pool. The flow cell was loaded onto a Nextseq 500 sequencing system (Illumina), and sequencing was performed with a 1 × 75 base pair read length. Tophat (v2.0.13) was used to align data to the reference rat genome (rn5), while Cuffdiff2 (v2.2.0) was used to identify DEGs.

### Pathway analysis

DEGs were selected according to two criteria: (1) > 2-fold change in expression with AOM exposure; (2) a false discovery rate (FDR)^37^ < 0.1. DEG pathway analysis was performed using the iPathwayGuide database^24^ (see http://www.advaitabio.com/ipathwayguide.html).

### mRNA expression analysis by RT-qPCR

Employing prepared cDNA, quantitative reverse transcription PCR (RT-qPCR) was performed using the LightCycler-DNA Master SYBR Green I Kit (Roche, Basel, Switzerland) according to the manufacturer’s instructions with an ABI7900HT Real-Time PCR System (Applied Biosystems). Measurement of gene expression was performed for target genes using rat-specific primers purchased from Exiqon (Vedbaek, Denmark). The relative quantity of the target mRNA was normalized to an endogenous gene (glyceraldehyde 3-phosphate dehydrogenase, GAPDH). The fold changes in RNA expression were calculated using the 2^−ΔΔCt^ method.

### ACF analysis

The number of Aberrant crypt foci (ACF) in the entire distal tubule was determined for animals in groups I1 to I6, as previously described^20^. Briefly, colons opened longitudinally were rinsed with PBS, and the rest of colon tissue excised by approximately 1 cm in width was positioned flat between two pieces of filter paper and fixed in 10% formalin for 24 h. Colons were then stained with 0.2% methylene blue in PBS for 10 min, placed on a microscope slide with the mucosal side up, and visualized using a light microscope (Olympus, Tokyo, Japan) at ×200 magnification. ACF were distinguished from normal crypts by an increased distance from surrounding cells, elongation of crypt size and morphology, and an easily discernible pericryptal zone. The total number of ACF in each colon and the multiplicity of AC in each focus were counted. ACF were categorized according to crypt multiplicity (1, 2, 3, or ≥ 4 crypts).

### Histological analysis of tumor mass

In order to analyze histological changes in colon polyps in groups M7 to M10, rats were sacrificed 1 week after the last acupuncture treatment, which was maintained for forty weeks following AOM treatment for tumor progression. All polyps were fixed in 4% neutral buffered formalin, embedded in paraffin, sectioned (4 µm thickness), and mounted onto glass slides. Hematoxylin-eosin staining (H&E) of tumor tissue was performed as previously described^38^. Tissue was observed using light microscopy (DFC425C, Leica, Wetzlar, Germany). Tumor size was measured using Leica Application Suite ver. 2.1.0.

### Expression of β-catenin by immunohistochemistry (IHC)

β-catenin expression in tissues without polyps was analyzed using IHC. A piece of tissue from the internal end of distal colon, approximately 1 cm wide, was collected from rats in groups M7 to M10. Half of each tissue sample was fixed in 4% neutral buffered formalin for 24 h, paraffin embedded and cut into 4 μm thick sections. IHC of colon tissues was performed by using an EnVision^TM^ Detection System (Dako, Troy, MI, USA), according to manufacturer’s instructions. Briefly, tissue sections were mounted onto silanized charged slides and allowed to dry for 1 h at room temperature, followed by 1 h in a 60 °C incubator. After deparaffinization and rehydration, slides were incubated with a proteinase K solution (20 μg/mL^−1^) for 5 min. After washing with distilled water, tissue sections were immersed in 3% H_2_O_2_ for 5 min to block endogenous peroxidase, followed by additional washing with buffer. Slides were incubated with a rabbit anti-β-catenin primary antibody (Abcam, Cambridge, UK) for 30 min in a humid chamber. After 2 rinses in buffer, slides were incubated with the detection system for 30 min. Staining was visualized with a 2,4-diaminobutyric Acid (DAB) substrate chromogen solution. Sections were dehydrated, mounted with cover glass, and observed using light microscopy (DFC425C, Leica, Wetzlar, Germany).

### Analysis of CRC marker (CEA, β-catenin) concentration using ELISA

CEA, a representative circulating biomarker for CRC, was analyzed in the serum of rats from groups M7 to M10. CEA level was determined using a rat CEA ELISA kit (LSBio, Seattle, WA, United States), according to manufacturer’s instructions. To determine β-catenin concentration in animals from each group, a piece of distal colon tissue without polyps, approximately 0.5 cm wide, was immersed in RIPA buffer (Sigma-Aldrich). Protein extracts were prepared according to manufacturer’s instructions. β-catenin concentration in the protein extracts was measured using a β-catenin ELISA kit (Enzo Life Sciences, Farmingdale, NY, USA).

### Statistical analysis

For all experiments, data from three independent experiments were analyzed using the Student’s *t*-test and are reported as mean ± SD. Sigma Plot version 12.3 (Systat Software, Inc., Chicago, IL, USA) was used to determine *p*-values. A *p* < 0.05 was considered statistically significant.

## Electronic supplementary material

Supplementary Information
