# Supplementary material for: Enhanced Therapeutic Treatment of Colorectal Cancer Using Surface-Modified Nanoporous Acupuncture Needles
Source: Sci Rep. 2017 Oct 10;7:12900. doi: 10.1038/s41598-017-11213-0 (PMC5635022; doi:10.1038/s41598-017-11213-0)
Supplement: Supplementary file 1 — Supplementary Information [file 41598_2017_11213_MOESM1_ESM.doc]

***Supplementary information for***

**Enhanced Therapeutic Treatment of Colorectal Cancer**

**Using Surface-Modified Nanoporous Acupuncture Needles**

Bo Ram Lee^1^, Hye-Rim Kim^2^, Eun-Sook Choi^1^, Jung-Hoon Cho^1^, Nam-Jun Kim^3^, Jung-Hee Kim^1^, Kyeong-Min Lee^1^, Abdul Razzaq^2^, Hansaem Choi^2^, Yunju Hwang^2^, Craig A. Grimes^4^, Bong-Hyo Lee^3§^, Eunjoo Kim^1^* & Su-Il In^2^*

*^1^Division of Nano & Energy Convergence Research, Daegu Gyeongbuk Institute of Science and Technology (DGIST), 333 Techno Jungang-daero, Hyeonpung-myeon, Dalseong-gun, Daegu, 42988, Republic of Korea.*

*^2^Energy Science & Engineering, Daegu Gyeongbuk Institute of Science and Technology (DGIST), 333 Techno Jungang-daero, Hyeonpung-myeon, Dalseong-gun, Daegu, 42988, Republic of Korea.*

*^3^College of Korean Medicine, Daegu Haany University, 136 Shincheondong-ro, Suseong-Gu, Daegu, 42158, Republic of Korea.*

*^4^Flux Photon Corporation, 116 Donmoor Court, Garner, NC, 27529, United States.*

*Corresponding authors for overall studies: Su-Il In, Ph.D. ([insuil@dgist.ac.kr)](mailto:insuil@dgist.ac.kr)) and Eunjoo Kim, Ph.D. ([ejkim@dgist.ac.kr](mailto:ejkim@dgist.ac.kr)).

^§^Corresponding author for acupuncture treatment: Bong-Hyo Lee, O.M.D. ([dlqhdgy@dhu.ac.kr](mailto:dlqhdgy@dhu.ac.kr)).

**Supplementary Table S1.** EDS (Energy Dispersive spectroscopy) data before and after anodization of conventional acupuncture needles, anodized at 20 V for 30 min.

|  | **Before Anodization (CN)** | | **After Anodization (PN)** | |
| --- | --- | --- | --- | --- |
| **Elements** | **Atom. C (at. %)** | **Error (%)** | **Atom. C (at. %)** | **Error (%)** |
| Fe | 40.46 | 1.69 | 26.03 | 1.39 |
| Cr | 12.00 | 0.51 | 8.01 | 0.44 |
| C | 42.71 | 3.06 | 63.24 | 4.70 |
| Ni | 4.32 | 0.28 | 2.46 | 0.22 |
| Si | 0.51 | 0.06 | 0.25 | 0.05 |

**Supplementary Table S2.** Total number of up- or down-regulated differentially expressed genes (DEGs) in each group compared with control group I1 (2-fold changes and false discovery rate [FDR] < 0.1).

|  | I2 | I3 | I4 | I5 | I6 |
| --- | --- | --- | --- | --- | --- |
| Up | 28 | 0 | 54 | 1 | 57 |
| Down | 18 | 21 | 15 | 10 | 100 |
| Total | 46 | 21 | 69 | 11 | 157 |

**Supplementary Table S3.** Effects of acupuncture treatment on pathways altered by azoxymethane (AOM) treatment in the initiation acupuncture treatment groups (I2–I6).

| Pathway | Significance of changes to control *(p*-value) | | | | | |
| --- | --- | --- | --- | --- | --- | --- |
|  | I2  (positive control) | I3  (CN/HT7) | I4  (CN/SI5) | I5  (PN/HT7) | | I6  (PN/SI5) |
| PPAR signaling | 9.8E-06 | 4.7E-02 | - | - | - | |
| AMPK signaling | 9.8E-06 | 1.2E-02 | - | - | - | |
| Adipocytokine | 3.5E-04 | -^a)^ | 9.0E-03 | - | 3.6E-02 | |
| Fatty acid metabolism | 2.0E-03 | - | - | - | - | |
| Fatty acid biosynthesis | 7.0E-03 | - | - | - | - | |
| Fatty acid degradation | 2.0E-02 | - | - | - | - | |
| NK cell mediated cytotoxicity | 3.0E-02 | 3.6E-02 | - | - | - | |
| African trypanosomiasis | 3.2E-02 | - | - | - | - | |
| Glycerolipid metabolism | 3.7E-02 | - | - | - | - | |
| Fat digestion and absorption | 3.7E-02 | - | - | - | - | |
| ECM-receptor interaction | 3.8E-02 | - | - | - | - | |

^a)^Calculated as *p* > 0.05.

**Supplementary Table S4.** Expression level of differentially expressed genes (DEGs) after azoxymethane (AOM) treatment, as analyzed by qPCR.

| Gene | I2 vs. I1^a)^ | I3 vs. I2 ^a)^ | I4 vs. I2 | I5 vs. I2 | I6 vs. I2 | Biomarker |
| --- | --- | --- | --- | --- | --- | --- |
| ACSBG1 | 0.68 ± 0.26 (0.020)^b)^ | 0.62 ± 0.30 (0.0040) | 0.22 ± 0.39 (0.12) | **-0.89 ± 0.50 (0.0017)** | -0.08 ± 0.54 (0.48) |  |
| IRF7 | 0.87 ± 0.71 (0.025) | 0.78 ± 0.36 (0.093) | 0.33 ± 0.47 (0.14) | 0.092 ± 0.40 (0.35) | 1.26 ± 0.27 (0.0013) | Neoplasia |
| SOD3 | 0.36 ± 1.01 (0.045) | **-0.80 ± 0.52 (0.031)**^c)^ | **-1.45 ± 0.36 (0.0022)** | **-1.80 ± 0.37 (9.0E-04)** | **-1.48 ± 0.62 (0.0035)** | ACF |
| ACS1 | 1.07 ± 0.87 (0.011) | **-0.84 ± 0.43 (0.048)** | **-1.22 ± 0.23 (0.013)** | **-1.16 ± 0.11 (0.014)** | **-1.84 ± 0.90 (0.0063)** |  |
| ADIPOQ | 4.48 ± 2.89 (0.028) | **-3.74 ± 0.76 (0.013)** | **-4.95 ± 1.55 (0.011)** | **-5.69 ± 0.46 (0.0095)** | **-3.97 ± 1.49 (0.015)** |  |
| ATF3 | -1.38 ± 0.36 (6.4E-11) | -0.36 ± 0.59 (0.11) | **0.67 ± 0.47 (0.0020)** | **0.68 ± 1.03 (0.0073)** | **1.35 ± 0.65 (6.0E-4)** | CRC |
| BECN1 | 1.69 ± 1.33 (0.006) | **-1.23 ± 0.71 (0.040)** | **-1.90 ± 0.35 (0.0082)** | **-2.26 ± 0.12 (0.0051)** | **-2.03 ± 0.52 (0.0073)** |  |
| CD36 | 1.63 ± 1.53 (0.013) | **-1.34 ± 0.33 (0.038)** | **-2.10 ± 0.38 (0.014)** | **-2.60 ± 0.13 (0.0092)** | **-2.36 ± 0.55 (0.012)** |  |
| CES1D | 2.06 ± 1.21 (0.0055) | **-1.71 ± 0.48 (0.013)** | **-1.95 ± 0.45 (0.0091)** | **-2.41 ± 1.24 (0.0090)** | **-2.39 ± 0.69 (0.0060)** |  |
| COL5A3 | 1.35 ± 0.52 (3.6E-04) | **-1.38 ± 0.63 (0.0011)** | **-1.53 ± 0.27 (2.8E-04)** | **-1.61 ± 0.16 (2.0E-04)** | **-1.49 ± 0.58 (6.3E-04)** | CRC |
| DGAT2 | 1.42 ± 1.09 (0.0079) | 0.57 ± 2.54 (0.23) | **-1.17 ± 0.14 (0.021)** | **-1.43 ± 0.038 (0.012)** | -0.48 ± 0.18 (0.13) |  |
| FABP4 | 2.36 ± 2.04 (0.012) | -0.56 ± 2.78 (0.075) | **-2.51 ± 0.60 (0.019)** | **-3.75 ± 0.14 (0.010)** | **-2.61 ± 0.73 (0.018)** |  |
| GPD1 | 1.93 ± 0.43 (1.2E-04) | **-1.17 ± 0.75 (0.012)** | **-0.92 ± 0.40 (0.0047)** | **-1.06 ± 0.20 (0.0012)** | **-1.24 ± 0.18 (6.2E-04)** |  |
| HBA1/2 | 1.63 ± 1.50 (0.031) | -1.35 ± 0.21 (0.069) | **-2.85 ± 0.49 (0.023)** | **-2.34 ± 0.40 (0.030)** | **-2.24 ± 0.93 (0.037)** |  |
| HBB | 1.40 ± 1.27 (0.022) | -0.48 ± 0.18 (0.20) | **-2.32 ± 0.49 (0.015)** | **-1.89 ± 0.45 (0.022)** | **-1.51 ± 0.88 (0.045)** |  |
| SCD1 | 1.05 ± 0.12 (3.3E-04) | -0.48 ± 0.98 (0.22) | **-0.76 ± 0.36 (5.9E-05)** | **-1.38 ± 0.54 (3.4E-06)** | **-1.19 ± 0.80 (0.0015)** |  |
| SCGB1A1 | 1.01 ± 0.97 (0.038) | **-3.8 ± 3.19 (0.016)** | -0.92 ± 0.27 (0.077) | -0.48 ± 0.54 (0.24) | **-1.24 ± 0.26 (0.043)** |  |
| SLC1A3 | 1.60 ± 0.52 (7.2E-05) | -0.096 ± 0.80 (0.41) | **-1.97 ± 0.74 (3.7E-05)** | **-2.12 ± 0.67 (1.8E-05)** | **-2.24 ± 1.04 (2.3E-05)** | CRC |
| TUSC5 | 1.78 ± 2.41 (0.013) | **-2.77 ± 1.10 (0.017)** | **-3.69 ± 0.89 (0.0096)** | **-4.71 ± 0.92 (0.0075)** | **-3.76 ± 1.17 (0.010)** |  |
| (p < 0.05) | 19 | 10 | 16 | 17 | 16 |  |

^a)^Relative expression level of each group expressed as -ΔΔC_T_,_G1_ or -ΔΔC_T_,_G2_.

^b)^*p*-value in parenthesis.

^c)^Expression changes that were significantly recovered by acupuncture treatment are in bold.

**Supplementary Table S5.** Effect of acupuncture on aberrant crypt foci (ACF) formation in the distal colon at the initiation stage of colorectal cancer (CRC).

| Group | AOM | Crypt multiplicity per foci | | | | Total no. of ACF/colon |
| --- | --- | --- | --- | --- | --- | --- |
|  |  | 1 | 2 | 3 | > 4 |  |
| I1  (n = 3) | - | 0 | 0 | 0 | 0 | 0 |
|  |  | 0 | 0 | 0 | 0 | 0 |
|  |  | 0 | 0 | 0 | 0 | 0 |
| **(Average ± SD)** |  | **0.0 ± 0.0** | **0.0 ± 0.0** | **0.0 ± 0.0** | **0.0 ± 0.0** | **0.0 ± 0.0** |
| I2  (n = 3) | + | 10 | 15 | 8 | 10 | 43 |
|  |  | 4 | 13 | 11 | 4 | 32 |
|  |  | 5 | 8 | 4 | 1 | 18 |
| **(Average ± SD)** |  | **6.3 ± 3.2** | **12.0 ± 3.6** | **7.7 ± 3.5** | **5.0 ± 4.6** | **31.0 ± 12.5** |
| I3  (n = 4) | + | 8 | 15 | 8 | 5 | 36 |
|  |  | 10 | 18 | 11 | 9 | 48 |
|  |  | 1 | 16 | 3 | 3 | 23 |
|  |  | 4 | 5 | 6 | 5 | 20 |
| **(Average ± SD)** |  | **5.8 ± 4.0** | **13.5 ± 5.8** | **7.0 ± 3.4** | **5.5 ± 2.5** | **31.8 ± 12.9** |
| I4  (n = 4) | + | 2 | 8 | 2 | 5 | 17 |
|  |  | 9 | 8 | 8 | 2 | 27 |
|  |  | 7 | 4 | 6 | 3 | 20 |
|  |  | 1 | 5 | 6 | 4 | 16 |
| **(Average ± SD)** |  | **4.8 ± 3.9** | **6.3 ± 2.1** | **5.5 ± 2.5** | **3.5 ± 1.3** | **20.0 ± 5.0** |
| I5  (n = 4) | + | 2 | 1 | 0 | 0 | 3 |
|  |  | 0 | 0 | 0 | 0 | 0 |
|  |  | 0 | 0 | 0 | 0 | 0 |
|  |  | 0 | 0 | 0 | 0 | 0 |
| **(Average ± SD)** |  | **0.5 ± 1.0** | **0.3 ± 0.5** | **0.0 ± 0.0** | **0.0 ± 0.0** | **0.8 ± 1.5**** |
| I6  (n = 4) | + | 13 | 0 | 2 | 1 | 16 |
|  |  | 9 | 20 | 12 | 7 | 48 |
|  |  | 7 | 5 | 4 | 2 | 18 |
|  |  | 1 | 1 | 5 | 4 | 11 |
| **(Average ± SD)** |  | **7.5 ± 5.0** | **6.5 ± 9.3** | **5.8 ± 4.3** | **3.5 ± 2.6** | **27.3 ± 17.9** |

**Significantly changed compared to I2, *p* < 0.01.

**Supplementary Table S6.** Occurrence and size of cancerous colon tumors, group M7.

| **Animal No.** | **Location** | **Diagnosis**^a)^ | **Size (mm)** |
| --- | --- | --- | --- |
| 1 | Proximal 1 | N |  |
| 1 | Proximal 2 | N |  |
| 1 | Middle | N |  |
| 2 | Middle | N |  |
| 2 | Distal | N |  |
| 3 | Middle | N |  |
| 3 | Distal | N |  |
| 4 | Proximal 1 | N |  |
| 4 | Proximal 2 | N |  |
| 4 | Distal 1 | N |  |
| 4 | Distal 2 | N |  |
| 5 | Distal | N |  |
| Total No. of colorectal tumor-bearing animals (%) | | 0 (0%) | |
| Total No. of polyps | | 12 | |
| Total No. of tumors | | 0 | |

^a)^N, normal tissue; Tu, tumor tissue.

**Supplementary Table S7.** Occurrence and size of cancerous colon tumors, group M8.

| **Animal No.** | **Location** | **Diagnosis**^a)^ | **Size (mm)** |
| --- | --- | --- | --- |
| 1 | Proximal | N |  |
| 1 | Middle 1 | N |  |
| 1 | Middle 2 | Tu | 4.9 |
| 1 | Distal | N |  |
| 2 | Proximal 1 | N |  |
| 2 | Proximal 2 | N |  |
| 2 | Middle | N |  |
| 2 | Distal 1 | Tu | 6.1 |
| 2 | Distal 2 | Tu | 2.6 |
| 2 | Distal 3 | N |  |
| 3 | Proximal 1 | N |  |
| 3 | Proximal 2 | Tu | 1.4 |
| 3 | Distal | N |  |
| 4 | Middle | Tu | 3.2 |
| 4 | Distal 1 | Tu | 2.1 |
| 4 | Distal 2 | N |  |
| 5 | Proximal | N |  |
| 5 | Middle 1 | Tu | 2.3 |
| 5 | Middle 2 | Tu | 6.0 |
| 5 | Distal 3 | N |  |
| 6 | Proximal | N |  |
| 6 | Distal 1 | N |  |
| 6 | Distal 2 | Tu | 2.2 |
| 6 | Distal 3 | Tu | 3.2 |
| 6 | Distal 4 | N |  |
| Total No. of colorectal tumor-bearing animals (%) | | 6 (100%) | |
| Total No. of polyps | | 25 | |
| Total No. of tumors | | 10 | |
| Total No. of lymphoid tissues | | 9 | |

^a)^N, normal tissue; Tu, tumor tissue.

**Supplementary Table S8.** Occurrence and size of cancerous colon tumors, group M9.

| **Animal No.** | **Location** | **Diagnosis**^a)^ | **Size (mm)** |
| --- | --- | --- | --- |
| 1 | Proximal 1 | N |  |
| 1 | Proximal 2 | N |  |
| 1 | Distal 1 | N |  |
| 1 | Distal 2 | N |  |
| 2 | Proximal | Tu | 4.6 |
| 2 | Distal 1 | Tu | 7.2 |
| 2 | Distal 2 | Tu | 5.3 |
| 3 | Proximal 1 | N |  |
| 3 | Proximal 2 | N |  |
| 3 | Middle | N |  |
| 3 | Distal 1 | Tu | 1.6 |
| 3 | Distal 2 | Tu | 4.8 |
| 4 | Proximal | N |  |
| 4 | Middle 1 | N |  |
| 4 | Middle 2 | N |  |
| 4 | Distal | N |  |
| 5 | Proximal | Tu | 0.7 |
| 5 | Distal | N |  |
| 6 | Proximal | N |  |
| 6 | Distal 1 | Tu | 2.4 |
| 6 | Distal 2 | Tu | 5.4 |
| 6 | Distal 3 | N |  |
| Total No. of colorectal tumor-bearing animals (%) | | 4 (67%) | |
| Total No. of polyps | | 22 | |
| Total No. of tumors | | 8 | |

^a)^N, normal tissue; Tu, tumor tissue.

**Supplementary Table S9.** Occurrence and size of cancerous colon tumors, group M10.

| **Animal No.** | **Location** | **Diagnosis**^a)^ | **Size (mm)** |
| --- | --- | --- | --- |
| 1 | Proximal 1 | N |  |
| 1 | Proximal 2 | N |  |
| 1 | Distal | N |  |
| 2 | Proximal | Tu | 2.5 |
| 2 | Distal | N |  |
| 3 | Proximal 1 | N |  |
| 3 | Proximal 2 | N |  |
| 3 | Distal | N |  |
| 4 | Proximal | Tu | 1.6 |
| 4 | Middle | Tu | 1.9 |
| 4 | Distal | N |  |
| 5 | Proximal 1 | N |  |
| 5 | Proximal 2 | N |  |
| 5 | Middle | Tu | 2.9 |
| 5 | Distal | N |  |
| 6 | Proximal | Tu | 1.2 |
| 6 | Middle | Tu | 1.6 |
| Total No. of colorectal tumor-bearing animals (%) | | 4 (67%) | |
| Total No. of polyps | | 17 | |
| Total No. of tumors | | 6 | |

^a)^N, normal tissue; Tu, tumor tissue.

**Supplementary Table S10**. β-Catenin concentration in the distal colon at the maturation stage of colorectal cancer (CRC).

| Group | No. | β-catenin (pg/mg protein) | Normal/Tumor |
| --- | --- | --- | --- |
| M7 (n = 5) | 1 | 297 | N |
|  | 2 | 588 | N |
|  | 3 | 268 | N |
|  | 4 | 491 | N |
|  | 5 | 762 | N |
|  | Average ± SD | 481 ± 206 |  |
| M8 (n = 6) | 1 | 2319 | N |
|  | 2 | 1446 | Tu |
|  | 3 | 2072 | N |
|  | 4 | 3401 | Tu |
|  | 5 | 36598 | Tu |
|  | 6 | 43277 | Tu |
|  | Average ± SD (*p*) | 14852 ± 19556 (0.061)^a)^ |  |
| M9 (n = 6) | 1 | 107 | N |
|  | 2 | 5263 | Tu |
|  | 3 | 159 | Tu |
|  | 4 | 18 | N |
|  | 5 | 25 | N |
|  | 6 | 5741 | Tu |
|  | Average ± SD (*p*) | 1886 ± 2806 (0.087)^b)^ |  |
| M10 (n = 6) | 1 | 345 | N |
|  | 2 | 662 | N |
|  | 3 | 153 | N |
|  | 4 | 87 | N |
|  | 5 | 30 | N |
|  | 6 | 65 | N |
|  | Average ± SD (*p*) | 224 ± 242 (0.0048)^b)^ |  |

^a)^t-test compared to M7

^b)^t-test compared to M8


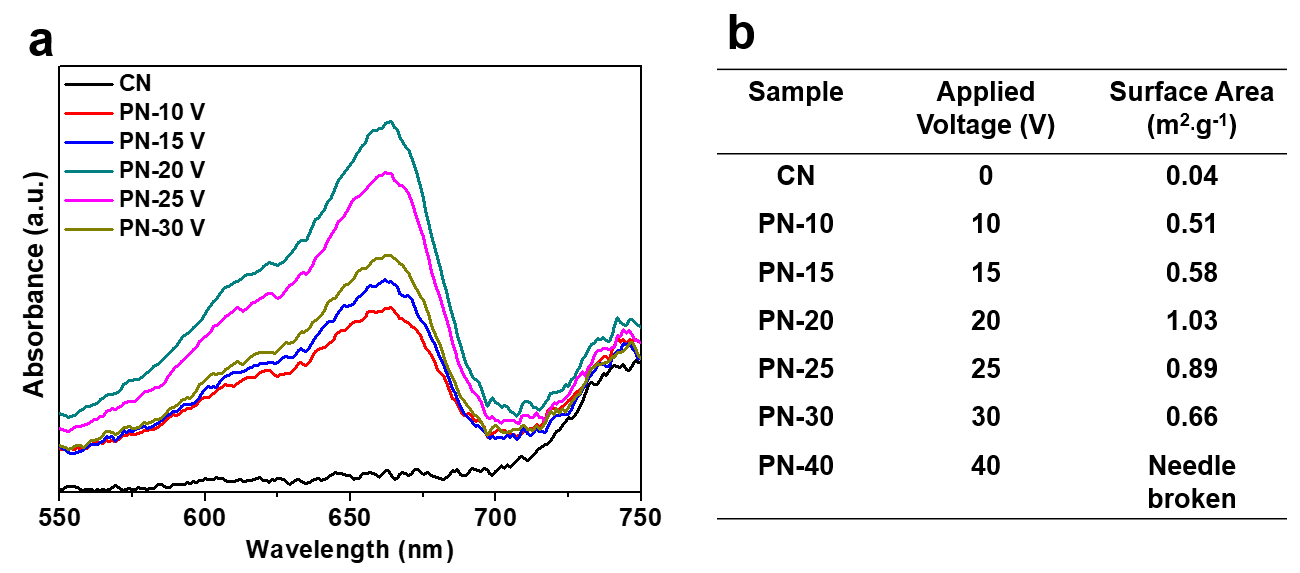


**Supplementary Figure S1.** **(a)** Absorption spectra for dye adsorbed on CN and various PN samples. **(b)** Surface areas determined for CN and various PN samples, the PN fabricated using 20 V anodization voltage shows a maximum result in both absorption spectra and surface area.


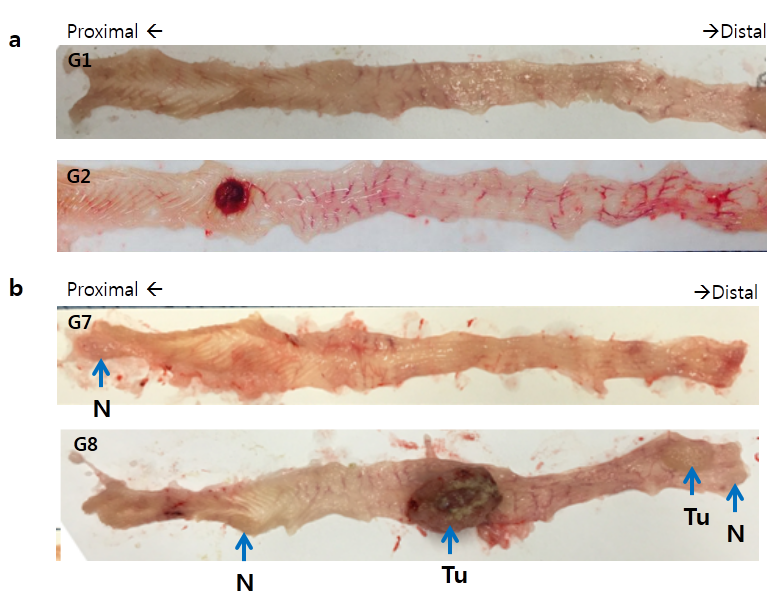


**Supplementary Figure S2.** Representative images of colons excised from sacrificed rats at **(a)** 6 weeks, and **(b)** 45 weeks following the AOM treatment.


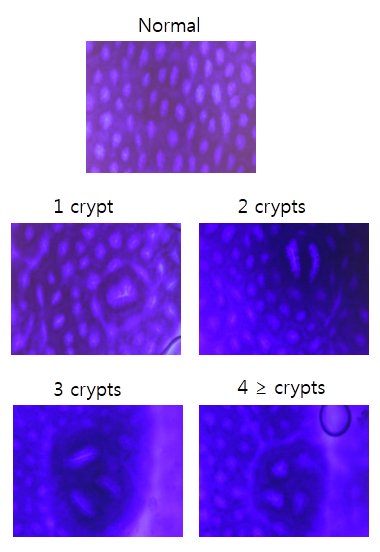


**Supplementary Figure S3.** Representative images of ACFs for each number of crypts. Images were captured by ×200 magnification.


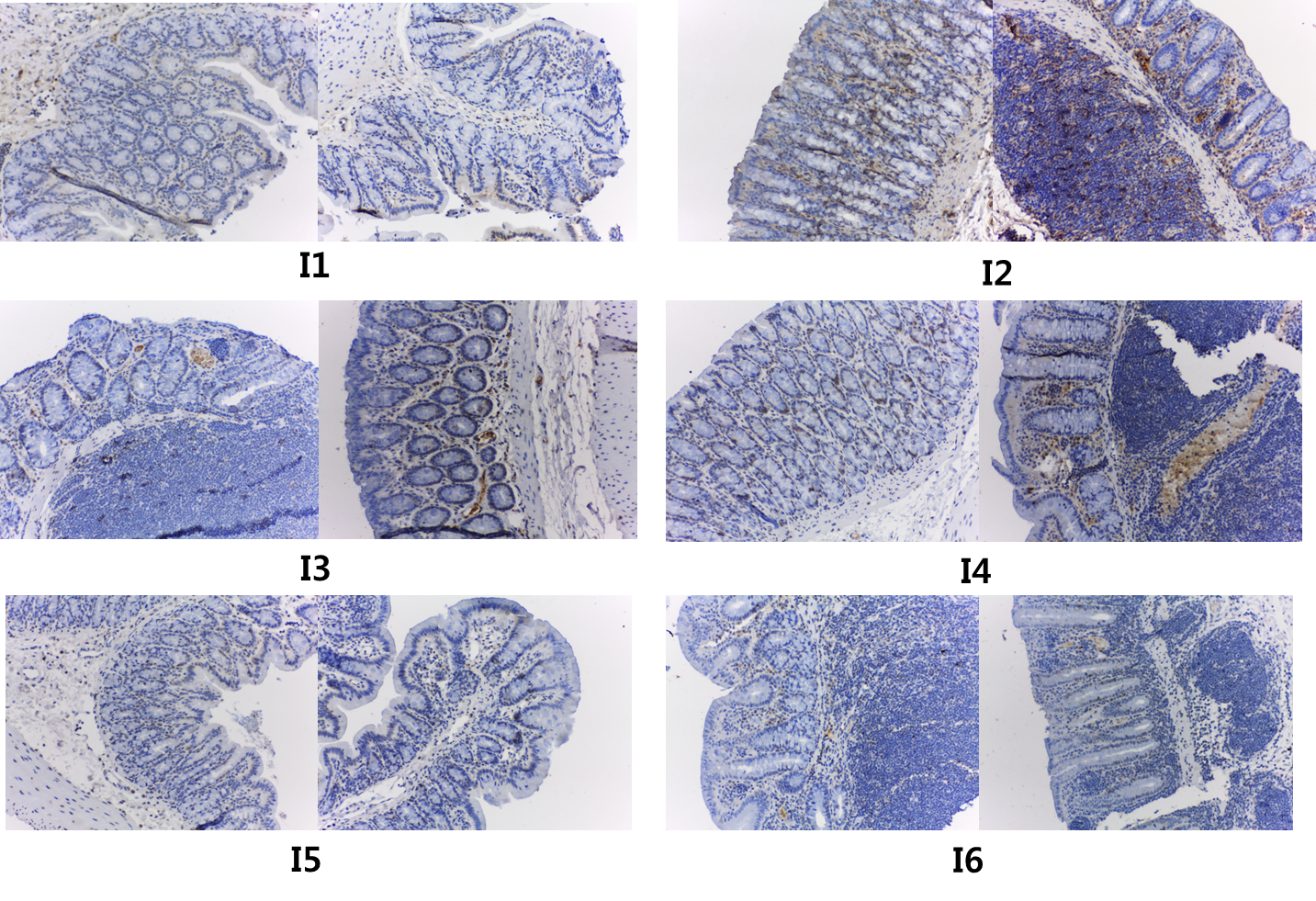


**Supplementary Figure S4.** Immunohistochemistry of β-catenin in the distal colon of initiation stage of CRC. Images magnified at ×200.


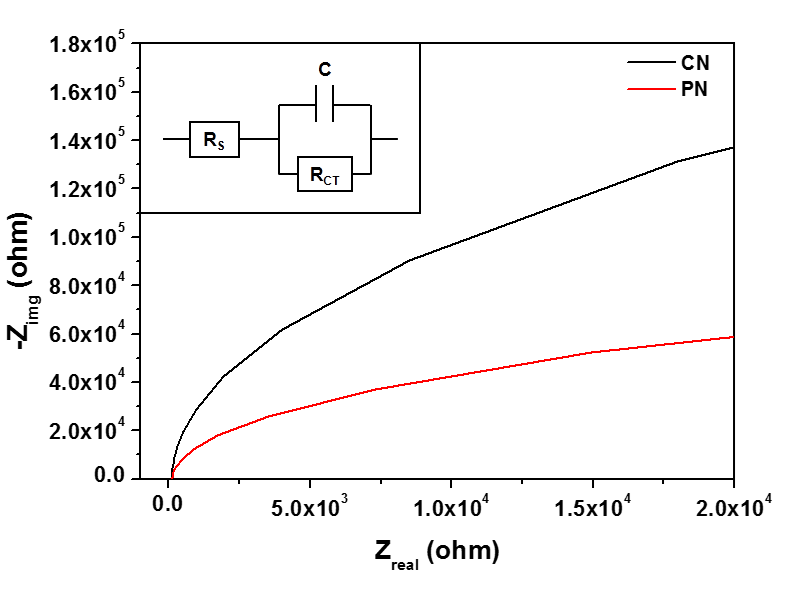


**Supplementary Figure S5.** Electrochemical impedance spectra (EIS) with fitted Nyquist plots corresponding to conventional acupuncture needle (CN) and nanoporous acupuncture needle (PN). The inset shows equivalent circuit for EIS measurement, where R_S_ = Solution resistance, R_CT_ = Charge transfer resistance and C = Double layer capacitance.
